# Dealing with pluralism: the managerial work of CEOs in Italian public healthcare organizations

**DOI:** 10.1186/s12913-022-08567-1

**Published:** 2022-10-01

**Authors:** Federico Lega, Andrea Rotolo, Marco Sartirana

**Affiliations:** 1 Dipartimento di Scienze Biomediche per la Salute and HEAD - Center for Research in Health Administration, Università degli studi di Milano, Milan, Italy; 2 CERGAS - Centre for Research on Healthcare Management - SDA Bocconi School of Management, Bocconi University, Via Sarfatti, 10, 20136 Milan, Italy

**Keywords:** Hospitals, CEOs, Managerial work, Pluralistic organizations, Italy

## Abstract

**Background:**

Healthcare organizations are extremely complex. The work of their CEOs is particularly demanding, especially in the public sector, though little is known about how the managerial work of a healthcare organization CEO unfolds. Drawing from scholarship on managerial work and management in pluralistic organizations, we sought to answer the questions: What is the content of managerial work of CEOs in public healthcare in Italy? How do healthcare CEOs perform their managerial work in complex interactions with multiple stakeholders?

**Methods:**

For this study we adopted a multi-method approach in which we conducted a survey to investigate CEO behaviors, tracked CEO working time for 4 weeks, and conducted semi-structured interviews with senior CEOs.

**Results:**

CEOs in public healthcare devote most of their time to interaction, which half of which is perceived as being occupied with apparently mundane problems. Nonetheless, devoting time to such activities is functional to a CEO’s goals because change in pluralistic contexts can be achieved only if the CEO can handle the organization’s complexity. CEOs do this by engaging in routines and conversations with professionals, creating consensus, and establishing networks with external stakeholders.

**Conclusions:**

CEOs are called to reduce fragmentation and foster cooperation across disciplines and professional groups, with the overarching aim to achieve integrated care. Using an analytical approach we were able to take into account the context and the relational dimension of the managerial work of healthcare CEOs and the specificities of this role.

**Trial registration:**

This article does not report the results of a healthcare intervention on human participants, and the material used in the research did not require ethical approval according to Italian law.

**Supplementary Information:**

The online version contains supplementary material available at 10.1186/s12913-022-08567-1.

## Background

### Introduction

Managing a healthcare organization is complex and the pressure on CEOs to run one efficiently is high. The scope of a CEO’s work is vast, the number of internal and external interlocutors potentially countless, and the constraints on effective strategic action numerous. A CEO faces internal pressure from professionals claiming autonomy and environmental pressure from external stakeholders with vested interests in influencing an organization’s policies and actions [1–3]. Because a public healthcare organization is complex, CEOs need to strike a balance between managerialism and professionalism, while being accountable to local politics and bureaucracy [4]. CEOs navigate such complexity in their daily managerial work.

The specificity of the role of CEO has been studied in the healthcare management literature from different perspectives. For instance, CEOs must possess numerous and complementary competencies in order to deal with the complexity of managing and reforming health care, which requires educational pathways [5]. Furthermore, experience is highly valued: CEOs with longer work experience in healthcare and in larger organizations are more likely to perform better, whereas the same does not hold true for mobility across industries [6]. Hospital performance is reportedly higher in those with CEOs who are doctors by training [7], as this strengthens their credibility with other physicians and enables them to enhance their competence in management and professionalism [3].

To our knowledge, no empirical studies to date have analyzed the daily work of healthcare CEOs, what they do in practice, and what type of activities take up their time. For this study we investigated what CEOs do on the job, who they interact with, and what such interactions entail. We draw from early research on managerial work such as Mintzberg’s [8] classical scholarship. With this approach, we wanted to explore the specific features – if any – characterizing managerial work in healthcare. Our first research question was “*What is the content of managerial work of executives in public healthcare?*”. We believe this is relevant, as previous research on managerial work has identified commonalities in managerial activity across time and industries, though studies on the work of healthcare CEOs are lacking.

Furthermore, we wanted to take a closer look at the complexity of a CEO’s work and the activities that take up most of their time and energy: how do they interact with stakeholders. To arrive at a contextual understanding of managerial work in a pluralistic healthcare organization, we asked ourselves “*How do healthcare CEOs actually perform their managerial work in complex interactions with multiple stakeholders?*”

To answer these questions we drew from the literature on managerial work and practice-based studies on management in pluralistic contexts. To do this we combined quantitative and qualitative approaches. Our focus was the work of CEOs in the Italian National Health System, as they are professionals (usually physicians) in charge of large healthcare organizations who exercise their strategic role in response to powerful internal and external stakeholder interests and political pressures within bureaucratic constraints.

Below we give a brief overview of the literature on managerial work and on executive management in pluralistic contexts. We then describe the study context and methodology, present our findings, and discuss the implications they may have for healthcare services.

#### The study of managerial work

The study of managerial work is well established in the organizational literature; the earliest reports date to Fayol [9]. Reflecting on his personal observations as a mining director, Fayol distinguished five functions of management: forecasting and planning; coordinating; organizing; commanding; and controlling. Building on this work, Gulick [10] coined the acronym POSDCORB for the main activities of a top manager: Planning, Organizing, Staffing, Directing, Co-Ordinating, Reporting, and Budgeting.

Henry Mintzberg [8, 11, 12] set the foundation for future research; his approach remains a major reference point [12]. Mintzberg [11] applied an empirical inductive approach to studying how top managers use their time. He critiqued the work of Fayol. After describing the actual morning routine of a top manager, he asked, “Which of these activities may be called planning, and which may be called organizing, coordinating, and controlling? Indeed, what do words such as ‘coordinating’ and ‘planning’ mean in the context of real activity? In fact, these four words do not describe the actual work of managers at all.” ([13]: 97). He directly observed a CEO’s activity for 1 week and analyzed the verbal contacts, the location of verbal contacts, the size of meetings, the participants attending the meetings, and the purpose of contacts. He found that, in contrast to theories on strategic programming, the activity of a CEO is highly fragmented, in which more time is spent on emerging day-to-day problems than on long-term issues. He also noted that CEOs devote a surprisingly greater amount of time to verbal than to written interaction and that almost half of meeting time was spent in encounters with subordinates, while the rest was devoted to superiors, co-directors, and external clients/suppliers.

Mintzberg ‘s work had a huge impact on later research [13]. Kurke and Aldrich [14] corroborated his findings in their study. Tengblad [15] returned to Mintzberg’s work to determine whether managerial work had undergone radical change over the decades in parallel with the evolution in managerial rhetoric towards non-hierarchical, more flexible forms of organization and greater emphasis on the top manager as a leader. He found that, while a relative shift in CEO behavior from administrative management to leadership can be envisioned, many of Mintzberg’s findings were still valid. The few exceptions were that a CEO’s work was less fragmented and interrupted, overall workload was heavier, time spent doing desk work was less, while more time was spent attending ceremonies. Similar results were found by Bandiera et al. [16] who collected data from a large sample of CEOs working in various countries and sectors. They found that CEOs spend 70% of their time interacting with others, especially with insiders (e.g., production and marketing staff), and 20% with outsiders (e.g., clients, suppliers, consultants).

Other authors [17] introduced a complementary dimension of analysis. They asked CEOs how much time they devoted to their core agenda and how much time in a reactive mode to handle unfolding issues, both internal and external, and in dealing with have-to-dos. They argued that CEOs should delegate operational tasks to managers and so free up time for activities on their strategic agenda. In contrast to Mintzberg, this implies that CEOs should focus on designing and implementing change. This opinion is shared by Kotter [18] who argued that CEOs should take a leadership role – cope with change - while delegating management – cope with complexity - to other members on the top management team.

These previous studies aimed at finding commonalities. Differences in epochs and approaches notwithstanding, rather similar patterns emerged about how CEOs allot their time to the tasks at hand. Despite their wide acceptance, these approaches were critiqued as they did not allow for a contextualized understanding of managerial work. Few exceptions aside (e.g., [19]), most studies examined managerial work separate from its institutional context and applied an analytical focus to individuals or jobs rather than relational practices [20]. Noordegraaf and Stewart ([21]: 440) underlined the theoretical and methodological shortcomings of these studies, urging the need to broaden and deepen the research approach and to study the “social embeddedness of managerial behavior”. Korica et al. [21] argued that, in order to bring “managerial work back to the future”, a turn towards practice approaches was necessary, shifting the analytical focus towards social relationships, discursive processes, and situated realities of everyday managerial work. Accordingly, they underlined the limitations of methods based on the quantification of activities and argued that such methods should be substituted or complemented with rich qualitative approaches that could explain a manager’s actions and interactions.

The theoretical and practical consequences of not doing so are relevant. For instance, in their study of four CEOs in Canadian public healthcare organizations, Johnson and Dobni [22] compared their findings to previous studies and argued that since high-level managerial work in the public sector is in many ways similar to that of the private sector, there is “evidence that indicates that managers may be transferrable across sectors … [and] that private sector managers can easily step into public roles due to parallels in tasks and responsibilities” ([22]: 467).

With the present study, we sought to couple this theoretical and methodological approach with the scholarship on managerial work in pluralistic organizations and thus grasp the specificities of public and of public professional contexts in particular.

#### Management in Pluralistic Contexts

Complex organizations contend with tension between powerful group interests, in which knowledge-based work predominates [2, 23]. All organizations are pluralistic to some degree. The highest level of pluralism is achieved when an organization strives to balance internal pressures from competing cultures and identities with external pressures through multiple and often contradictory strategies in response to conflicting environmental demands [23].

Such tensions result in difficult positions for executives. Top managers in a pluralistic context must couple leadership, organization, and environment to drive change [2]. They seek to find complex solutions often under enormous pressure on their time and attention, which may overstretch their capacity [23]. Solutions can be identified to meet these conflicting demands. Executives who are professionals themselves have the potential to take on a hybrid role [24, 25] in which they can more easily reconcile professional claims and managerial imperatives. Also, they can devote time to organize collective leadership constellations [26], i.e., top management structures in which individuals combine their expertise and source of legitimacy to deal with organizational complexity effectively.

As concerns their managerial work, CEOs foster participation by a wide range of actors in decision-making processes through a collaborative approach that involves stakeholders in navigating such complexity [27, 28]. Denis et al. [2] describe three complementary dimensions of effective management and strategy making in pluralistic organizations: nurture relationships to gather and mobilize tacit knowledge over time, find a synthesis between competing value systems to achieve legitimacy, and gain support from multiple actors by building networks that distribute power. The three dimensions are grounded on various different theoretical perspectives: social practice theory, conventionalist theory, and actor-network theory. Other examples of managerial actions that effectively address pluralism are: maintain frequent and close contact with stakeholders and grant them recognition, ensure that their demands are met, and set fragmented interests within a wider organizational context [28].

The concept of a pluralistic context is particularly appropriate for understanding the work of executives in the public sector and especially in public healthcare organizations owing to their characteristically high levels of pluralism [2, 29]. The CEOs of public healthcare organizations must deal with complex professional, administrative, and managerial cultures. They interact with powerful professionals [30] belonging to diverse subcultures of medical specialties that claim a legitimate stake in the formulation and implementation of strategies to advance their goals. Though CEOs cannot exercise hierarchical power over professionals, they do need to align diverging goals, manage dissent and turf wars, and contend with power plays in their interactions [31], all the while exercising political skills [25]. In this respect, being a hybrid professional can help in mediating diverse cultures and contrasting expectations [26]. One among the many internal challenges that public healthcare CEOs face is how to balance professional interests. Added to this are the myriad rules and regulations and the pervasive bureaucracy embodied by administrative staff [32, 33]. Summarizing, CEOs strive to reconcile professionalism, bureaucracy, and managerial logics and practices typical of new public management reforms [34].

Moreover, CEOs also come under strong external pressure. As reported by Lega [4], the CEOs in public healthcare organizations often receive vague, conflicting objectives from politicians and policymakers. The margin of autonomy that executives can exercise within political constraints is hard won from negotiation and well-managed relationships with key stakeholders, such as public officials and policymakers. CEOs need to maintain close contact with a variety of external stakeholders (e.g., local communities, media, private sector representatives), whose demands influence strategy and arouse ambiguity and uncertainty [31, 35].

In line with these studies, we combined in the present study the traditional research approach to managerial work with a more practice-based methodology to bring relationships and context into focus. Our analysis of the work of CEOs takes into account the specificities of the public healthcare sector and the challenges of interacting with a plurality of stakeholders.

## Methods

### Setting

The present study reports the findings from an analysis of the managerial work of CEOs in public healthcare organizations in Italy. The Italian National Health Service (NHS) is operated through twenty regional governments and coordinated by the Ministry of Health. The regional governments define the health policies and assign to public and private providers the goals and targets of outputs and/or clinical outcomes. There are three types of public healthcare organizations: local health authorities for the delivery of hospital care, primary care, and public health services; hospital trusts that deliver hospital care (some are also teaching hospitals); and research hospitals. A public healthcare organization will have, on average, about 1100 beds and over 3000 employees [36]. The CEOs heading these organizations are appointed by the regional governor based on a spoils system, a mechanism through which the regional government appoints them on a fiduciary basis. Their performance is evaluated based on annual targets set by the regional government and their mandate cannot exceed the regional governor’s mandate of 5 years; the length of a CEO’s mandate is 3.7 years on average [36]. More than two thirds of CEOs are physicians, most of which are specialized in public health and hospital management. This is considered helpful for understanding clinical issues and interacting with other professionals [37]. The CEO is supported by the organization’s medical director and administrative director (this triad is usually referred to as the strategic board), a director for social care, and a nurse director in some organizations.

### Methodology

A variety of methodologies have been developed to study managerial work, from in-depth observational analysis of a few cases to large-scale quantitative analysis of the agenda of CEOs. Depending on the research objectives, the approaches opt for diverse solutions to the trade-off between sample size and depth of research. For the present study, we built on the existing literature and adopted a multi-method framework with a three-step approach to gain a comprehensive understanding of the distribution of working time and the type of activities that CEOs carry out in a workday.

### Quantitative data collection

To do this, we developed a survey tool to measure CEO behaviors derived from two sections of the tool described in Bandiera et al. [16], who recorded the type of managerial activity based on diary entries of CEOs which they accessed via phone calls with the CEO or their personal assistants. The dimensions in the survey and the response options were defined a priori as: Type of activity (meeting, conference call, business lunch; individual work; public events/ceremonies; travelling; continuing professional education; personal/family) and Type of interlocutor in a meeting. Also, based on our experience in the Italian NHS, we identified the main internal and external interlocutors who interact with CEOs: regional government administrators, CEOs from other public healthcare organizations, administrative/medical directors, administrative staff, healthcare professionals, local government administrators and community leaders, trade union representatives, patient associations, industry, press/media, other (university, etc.).

The tool was tested with two CEOs and revised according to their feedback. We then asked executive assistants of the CEOs to track their boss’s work time in 30-minute segments, 7 days a week for 4 consecutive weeks (in March 2019), retrieve the data from their boss’s agenda, and then check data accuracy with their boss. The questionnaire was sent to the CEOs (*n* = 194) of Italian public healthcare organizations. The response rate was 20% (38/194 from nine regions), which we deemed appropriate, given the difficulty in accessing CEOs and the effort required of them and their assistants. The sample was 20 CEOs of independent, large public hospital trusts (including teaching hospitals) and 18 CEOs of local health authorities, including secondary care facilities. Most of the organizations were located in northern Italy (79%), had on average 800 beds, 3800 employees, and a turnover of €600 million (approximately US $720 million). Appendix A presents the details of the survey tool.

We followed up the first questionnaire with a second questionnaire to the 38 CEOs 1 month later in which they were asked to verify the responses to the first questionnaire (a table showing the tracking by the assistants for each CEO was made available) and to indicate the amount of time they worked that was not accounted for on their business agenda or calendar (e.g., night or weekend works). In line with Porter and Nohria [17], we asked the CEOs to specify how much time they spent in meetings with interlocutors discussing their organization’s strategic agenda and how much time they devoted to handling unfolding issues and dealing with have-to-dos (see Appendix A for details). Of the 38 CEOs contacted, 17 (45%) responded. The sample can be considered representative since the CEOs who responded to both questionnaires had held a CEO position also in other organizations (5.7 years and 5.5 years work experience, on average, for the first and the second questionnaire respondents, respectively) or was a member on a strategic board in a public healthcare organization (10.4 years and 11.3 years work experience, on average, for the first and the second questionnaire respondents, respectively). Male CEOs made up 85% of the respondents of the first questionnaire and 88% of the second; the average age was 58.5 and 58.6 years, respectively. The sample characteristics are consistent with a previous study on the curricula of healthcare CEOs in Italy [38] where the average age of a CEO was 59 years, the percentage of male CEOs was 85%, and the average time on a strategic board in a healthcare organization was 11 years. Table 1 presents the characteristics of the two samples.

**Table 1:** Sample characteristics

Characteristic	Questionnaire 1 (*N* = 38)	Questionnaire 2 (*N* = 17)
Average age -years	58.6	58.5
Male CEOs – percentage	84	88
Average number of years as CEO in the current organization	2.4	3.1
Average number of years as CEO in a healthcare organization	5.7	5.5
Average number of years as a member of a strategic board in a healthcare organization	10.4	11.3

### Qualitative data collection

We expanded the analysis to gain a more in-depth and nuanced understanding of the managerial work of executives working in large public professional healthcare organizations. To do this, we conducted interviews with ten experienced senior or retired CEOs (who had served in multiple healthcare organizations [3.7 organizations, on average, in different regions of Italy]) in which we asked them to comment on the overall survey findings and to provide a narrative account of the time they spent with internal and external interlocutors. The idea of interviewing a senior or a retired CEO (this sample differed from the two samples that responded to the questionnaires) was to obtain an external perspective on the data we collected and to check whether the questionnaire responses were consistent with the experience of top managers who had significant experience in top positions in a public healthcare organization. The interviews were conducted either in person or online and were arranged after sending the interviewees a summary of the questionnaire findings. In this way, we applied a multi-method triangulated approach to study the same phenomenon from multiple perspectives (executive assistants of CEOs, CEOs themselves responding to the second survey wave and the object of our study, and experienced CEOs through individual interviews as key informants). This was done to enrich our understanding of the topic and allow for more subtler dimensions to emerge. The study was designed to ensure that different viewpoints could converge (or diverge) and thus lead to an enriched explanation of the research questions [39].

Based on our theoretical framework and the literature on pluralistic organizations, we made a list of open-ended questions that were refined in a pilot interview. Respondents were asked to report on how CEOs balance the management of unfolding issues and their focus on a strategic agenda while interacting with main stakeholders. To increase data credibility, we encouraged the interviewees to give concrete examples from their professional experience. In this way, we were confident the responses were trustworthy [40]. Also, we asked the interviewees whether, based on their experience as a CEO in a healthcare organization, they agreed with the questionnaire findings. The interviews were conducted in Italian, consent to participate was obtained from all participants prior to the beginning of the study, and participant anonymity and confidentiality were guaranteed. The interview content was summarized to synthesize the findings [41].

### Qualitative data analysis

**Fig. 1:**
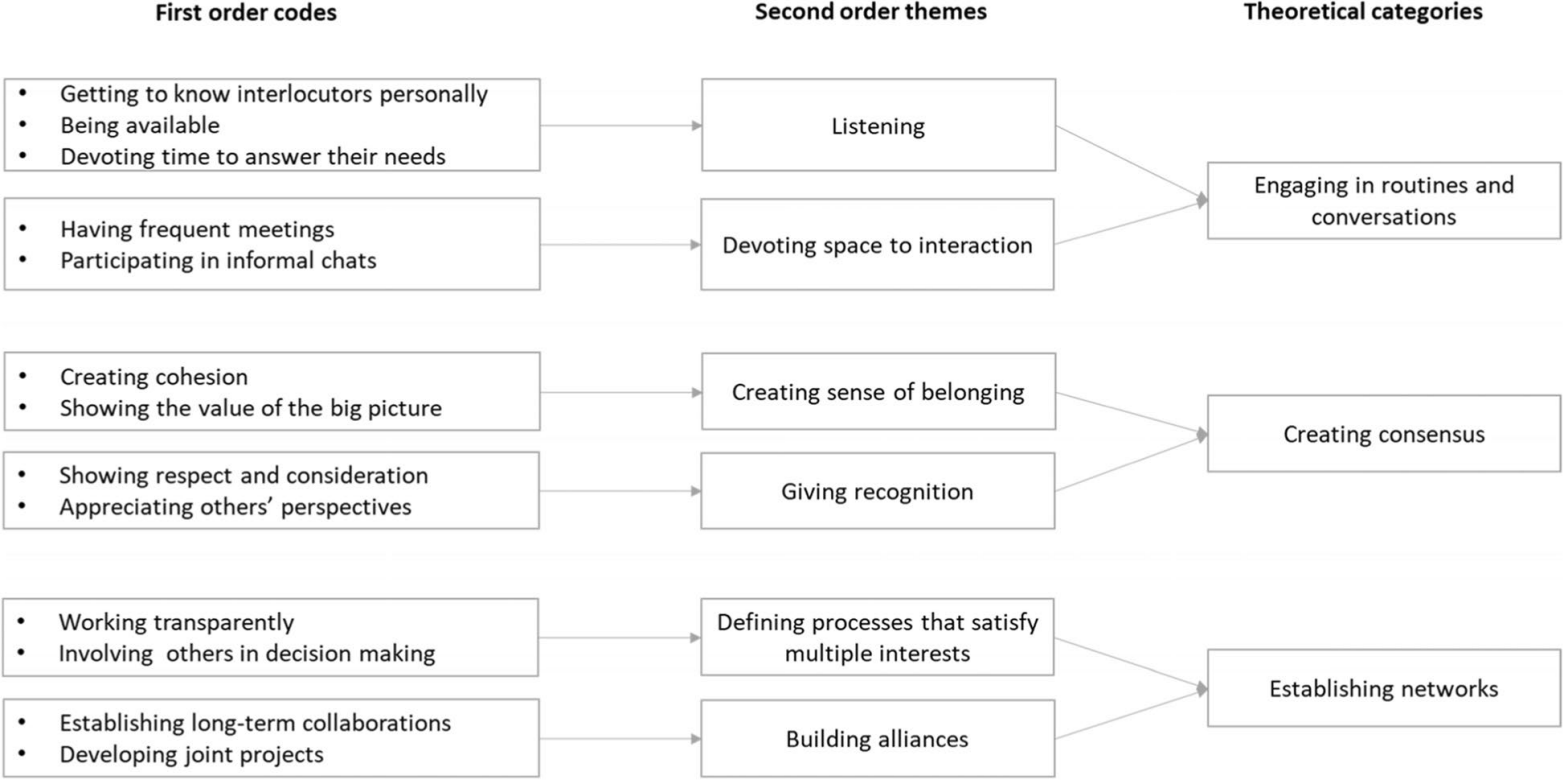
Data structure

We employed abductive reasoning [42, 43] to promote dialogue between empirical findings and theory in an analytical strategy based on iteration of questions and answers from previous studies in the literature and vice versa [44]. Initial conceptualization of the qualitative data was done by identifying relevant concepts in the empirical material using the CEOs’ perspectives as a starting point for the analysis and a constant comparison technique [45, 46]. One of the three authors identified thirteen different first-order codes, which were then aggregated into six second-order themes discussed with the other authors. We were guided in making sense of the second-order themes by scholarship on managerial work in pluralistic organizations. We derived three theoretical categories from the work of Denis and colleagues [2] on the three interconnected components of strategy making in pluralistic contexts: 1) engaging in routines and conversations; 2) developing a compromise among competing values (which we labelled creating consensus); and 3) establishing networks. For instance, first-order codes “showing the value of the big picture” or “creating cohesion” were aggregated in the second-order code “creating sense of belonging”, which refers to the aggregate category “creating consensus”. Similarly, first-order codes “working transparently” and “involving others in decision making” were aggregated in the second-order code “defining processes that satisfy multiple interests” and associated with the category “establishing networks”. The data structure is presented in Fig. 1.

## Results

### Quantitative analysis

Analysis of the diary entries showed that the healthcare CEOs were engaged in different types of activities for 50.3 hours on average per week. When we exclude the time tracked as dedicated to private commitments during regular working hours and add the number of hours of additional work performed at home (5.5 hours per week), the CEOs spent a total of 52.7 hours per week on managing their healthcare organization. More than half of the time (56% or 28.3 hours per week) was spent in face-to-face meetings. Some 23% of the time (11.5 hours per week) was devoted to individual work (e.g., preparing documents, reading, e-mail), while participation in public events (e.g., conferences, workshops, inaugurations) took up 8% of total time, 5% was spent travelling for work, and 2% in continuing professional education.

The data from the diary entries disclosed a more detailed picture of how the CEOs spend their time in meetings and interactions: 2.7 hours per week (5% of total time) spent with regional government administrators, CEO superiors, and 6.4 hours per week in interaction with other members of top management (strategic board members including administrative directors and medical directors), CEO co-leaders. Most meetings were with internal stakeholders: 7.2 hours per week (14% of total time) spent in discussions with healthcare professionals working for the organization, 6 hours (12%) in meetings with staff members of the organization’s auxiliary services (i.e., administrative staff and other managerial or administrative support roles), and 1.2 hours (2%) with union representatives. As regards external stakeholders, 1.6 hours (3%) were spent with local government administrators and 1 hour (2%) with CEOs from other healthcare organizations. Interaction with representatives from patient associations, media, pharma and medical technology companies, and other stakeholders took up a smaller proportion of time. Table 2 presents the number of hours spent in activities per week.

**Table 2:** Weekly number of hours healthcare organization CEOs spend on activities and with interlocutors (*N* = 38)

Activity	Hours/week	% of time
Meetings	28.3	56
* With regional government (superiors)*	*2.7*	*5*
* With strategic board members (co-leaders)*	*6.4*	*13*
* With internal stakeholders*	*14.4*	*28*
Professionals	7.2	14
Organizational technostructure	6.0	12
Union representatives	1.2	2
* With external stakeholders*	*4.9*	*9*
Local governments	1.6	3
Other CEOs	1.0	2
Patient associations	0.7	1
Media / press	0.6	1
Pharma and MT^a^ companies	0.2	0
Other stakeholders	0.8	2
Individual work (tracked in the diary)	11.5	23
Public events	4.1	8
Business trips	2.4	5
Continuing professional education	1.0	2
Family and private life (tracked in the diary)	3.0	6
Individual work (not tracked in the diary)	5.5	
**Total**	**52.7**	**100**

^a^Medical Technology

We then asked the CEOs to estimate the time they spent in meetings they perceived as being directly related to their strategic agenda and how much time they perceived spent in more operational issues. Table 3 shows that just over half of the time in interaction with stakeholders was perceived as being related to the strategic agenda, while a vast portion was considered operational.

**Table 3:** Strategic relevance of meetings (*N* = 17)

Meetings with	% perceived as part of the strategic agenda
Regional government administrators	76
Strategic board members	69
CEOs from other healthcare organizations	63
Local communities	58
Union representatives	52
Patient associations	50
Organizational ancillary services	49
Healthcare professionals	48
Pharma and medical technology companies	48
Press / media	36
Other stakeholders	42
**Total**	**56**

### Qualitative analysis

In a final step we carried out interviews with experienced senior or retired CEOs who assisted the research team in sense-making of the responses and in analyzing the role of CEOs in public healthcare organizations. Our rationale was that an understanding of how they interact with internal and external stakeholders can reveal how managerial work is actually performed. We asked the interviewees to tell us about how they performed their managerial activity, while balancing the time devoted to unfolding issues and to their strategic agenda. We found that the CEOs devoted most of their time and effort to engaging in routines and conversations with internal and external stakeholders, to creating consensus, and to establishing networks. As anticipated, we found that the analytical framework developed by Denis et al. [2] regarding strategic actions of executives in pluralistic organizations best explains our empirical data.

Firstly, achieving and distributing knowledge by engaging in routines and conversations. The interviewees reported how important it was to listen to stakeholders, especially other professionals, to obtain information on the organization, where problems are arising, which needs must be addressed, and which challenges lie ahead. The interviews also disclosed that CEOs devote considerable space to interaction by engaging in informal conversations and frequent meetings which, while seemingly operational or mundane, demand prompt attention to prevent them from becoming larger issues.

*“I personally managed the budget meetings to get to know professionals, to understand the internal dynamics.”* (CEO #7)

*“Sometimes I call them, but very often they are looking for me. I often meet unit directors and professors because I want to hear how the hospital is going, and I don't want my knowledge to be filtered by my staff. This is not really separate [from strategy making], an informal exchange with a professional can have strategic significance.”* (CEO #6)

*“Professionals must be pampered and stimulated, the relationship with them is fundamental and takes a lot of hours. You need to talk to professionals about data on a monthly basis, at least with the most important ones.”* (CEO #8)

Secondly, finding a viable compromise among competing values by creating consensus**.** A centrally important aspect of a CEO’s managerial activity is maintaining a relationship with stakeholders in which a sense of belonging is created by showing the value of big picture. This is vital to facilitate collaborative approaches, especially among internal stakeholders, first and foremost the professionals, and to reduce fragmentation typical of pluralistic contexts. Furthermore, the CEOs invest in giving recognition, by appreciating the stakeholders’ perspective and showing them consideration.

*“Professionals see only their specialty … but do not see themselves as part of the broader care process. I work on the sense of belonging … and on creating a shared vision … today the greatest amount of time of a CEO is spent in relationships, with professionals and their spokespersons, generally trade unionists. … everyone is entrenched, protects their fort, and the CEO must put him in a net: this is the challenge that occupies 40% of my work.”* (CEO #4)

*“I often observe that they simply need to be listened to and they need recognition, and I think it is appropriate to dedicate good part of my time in this activity, which can foster a good corporate climate and nurture a sense of belonging to the group.”* (CEO #7)

Thirdly, executives also work to build networks with stakeholders to manage power and interests**.** Internally this refers to defining the processes that satisfy multiple stakes: professionals must be involved in decision making to overcome resistance to change and reduce the distance between professionals and administrative staff and across professional subgroups. But it is also important for external stakeholders: as in Italy there is no formal committee devoted to building relationships with the community, as reported for other countries (e.g., [1]), the CEO himself is called to build alliances by developing long-term collaboration and joint projects with politicians, local government, patient associations, and other representatives of civil society.

*“Frequent relationships with both formal representatives of the communities and the citizens’ groups [are important] in order to explain how much they are involved in the strategies [of the hospital] and in the technical choices made to pursue them. Direct confrontation, transparency, and consistency have more often yielded positive results than avoiding the encounter.”* (CEO #9)

*“Relations with mayors and associations are fundamental, perhaps they are the most important ones: we need to increase forms of ‘alliance’, for example by organizing joint events: conferences, training events, information campaigns..* (CEO #1)

*“Patient organizations are important partners and sometimes the CEO has to be there to meet them, especially the categories more in need or more relevant in the area (e.g., paraplegics, dialysis patients), otherwise situations that could be avoided explode because of nothing.”* (CEO #3)

Table 4 summarizes the results of the qualitative analysis and the content of managerial work, the main activities and the interlocutors involved.

**Table 4:** How CEOs interact with stakeholders

*Managerial work*	*Focus*	*Activity*	*Stakeholders*
Engaging in routines and conversations	Knowledge	• Listening: getting to know interlocutors personally, being available, devoting time to respond to needs • Devoting “space” to interaction: frequent meetings, participating in informal chats	Mostly internal
Creating consensus	Values	• Creating a sense of belonging: creating cohesion, showing the value of the big picture • Giving recognition: showing respect and consideration, appreciating others’ perspectives	Mostly internal
Establishing networks	Power and interests	• Defining processes that satisfy multiple interests: working transparently, involving others in decision making • Building alliances: establishing long-term collaboration, developing joint projects	Mostly external

## Discussion

Our findings answer the research questions about what characterizes the managerial activity of CEOs in pluralistic contexts by showing how they perform managerial work while dealing with complex interactions with stakeholders. We identified several differences compared to previous studies on CEOs in the private and the public sector. One was the average work week length of 52.7 hours which, albeit not significantly different from Johnson and Dobni [22], was shorter than what most studies report for the private sector. Also, traveling time was far shorter than reported elsewhere probably because of the smaller catchment area of Italian public healthcare organizations compared to large companies in other industries.

Our findings are consistent with previous studies on the proportion of time allocated to activities, the amount of time spent in meetings and interactions, and how much interaction time is allotted to diverse interlocutors, especially compared to the studies by Mintzberg and Tengblad [11–13, 15]. Like Johnson and Dobni [22], we noted that interaction with external stakeholders did not figure prominently on the CEO agenda. A few minor differences appeared, however. For instance, in their study of public healthcare CEOs, Johnson and Dobni [22] found that much more time was spent in interaction with clinicians than we did (14% versus 8%) and time spent with superiors.

Nearly half of CEO time was perceived as not being directly related to a strategic agenda but rather to operational apparently mundane have-to-do tasks. At first sight, this could be read as a signal that CEOs might do better by delegating such tasks to co-leaders and so free up time for strategic activities and steering the organization. Previous studies suggest that CEOs should focus their energy on bringing about change in the organization and devote more time to advancing their own agenda by delegating the management of complexity and operational tasks [17].

We found, however, that in pluralistic contexts the managerial work of a CEO is by and large exercised through this latter type of activities. In our sample, the CEOs stated that they nurtured relationships with key stakeholders and invested time in listening to their needs often in informal ways; in doing so they felt better able to identify points of convergence across different value systems and foster consensus; and they worked to build alliances to align diverging interests. They were aware that they had to be ready to handle urgent though apparently insignificant problems which, if not promptly addressed, might become more serious or compromise relationships. Summarizing, change in a pluralistic context can be effectively achieved by dealing with its complexity. The time and energy that CEOs invest in daily interaction with stakeholders serve to gather information, build consensus, and create networks for establishing collaborative decision-making processes that ultimately drive change.

The three components of managerial work combine in complementary dimensions. When engaging in routines and conversations, CEOs focus on the acquisition and distribution of tacit knowledge. Personal contact is extremely relevant in professional organizations when plans and strategies of hospital department heads, as well as quality or coordination problems, need to be identified. Not surprisingly, this approach is also key to interacting with administrative staff when rules and regulations are pervasive, as CEOs need to find effective and sometimes creative solutions to avoid being entrapped by bureaucracy.

Moreover, healthcare CEOs often contend with competing values. They strive to balance professionalism, managerialism, and compliance with regulations and public accountability by engaging internal organizational constituencies, i.e., the professionals and the administrative staff embodying them. A similar objective is followed to settle turf wars between competing professions and across medical specialties within the organization. Finally, CEOs exert power by connecting interlocutors and by negotiating objectives with politicians and policymakers and with other external interlocutors.

Our qualitative analysis revealed that Italian healthcare CEOs perceive networks’ management as a key activity in their mandate. The time they devote to building and nurturing relationships with internal and external stakeholders takes priority on their agenda. Nearly all interviewees stated that this time investment was relevant and essential to create the conditions for carrying through with their strategies. When talking about their relationship with the professionals working inside the healthcare organization, the interviewees described how professionals, by virtue of their role, pursue individual goals and principles. The CEOs realize that the time spent in meetings is the only way they have to persuade the professionals to adopt a shared vision and align their objectives and values with those of the organization.

This is coherent with the complexities characteristic of the healthcare sector and of the Italian NHS in particular, where there is an urgent need to reduce fragmentation and foster cooperation across specialties and professional groups to develop care pathways and integrated care within organizations and externally among stakeholders who represent the needs of patients [34]. This is all the more relevant in the context of public healthcare, where CEOs work under the constraints of organizations dominated by a bureaucracy in which a better understanding of the regulatory and legal configurations of a problem could result extremely effective.

Moreover, by building on the literature, we adapted and developed a quali-quantitative methodology to measure how healthcare CEOs use their time. This was done to answer the calls [21] to take into account the context of managerial work and its relational dimension and to broaden and deepen our analytical approach to studying the social and institutional embeddedness of managerial work [20]. Our quantitative findings share many similarities with previous studies (e.g., [15, 17, 22]). We believe, however, that a complementary qualitative analysis that can capture managerial work and the strategic dimension of interaction with stakeholders allowed to record the relevant differences between contexts. Using this approach we can better understand what top management in public healthcare is really like, the commonalities it shares with other sectors, and in what ways it differs from them. We argue that our analysis describes the features of a CEO’s managerial work in a pluralistic context like public healthcare. Our findings contrast with those of Johnson and Dobni [22] who claimed that similarities prevail and that top managers may be transferrable across sectors. Differently, we found specificities of managing public healthcare organizations. The distinct competencies, skills, and attitudes that CEOs need to carry out their managerial tasks may preclude transferability across sectors. This is in line with previous studies that emphasized the importance of sector-specific work experience rather than mobility across industries [6] and with studies on doctors or professional hybrids heading healthcare organizations [3, 7].

The present study has several limitations. Although our response rate was acceptable, given the effort requested of the CEOs and their assistants, the sample is relatively small. Larger samples may better control for contextual factors that might impact on time allocation. Furthermore, the CEOs and their assistants were asked to describe the distribution of their time for 1 month. We are aware of possible seasonality in time allotment and of the influence of the phase of a CEO’s managerial mandate. A longer observation period could yield more insight into the characteristics of managerial work by CEOs in public healthcare organizations. Our data were collected from CEOs working in public healthcare organizations in the Italian NHS, while a more detailed picture might be captured by studying executives working in the healthcare systems of other countries. Comparative research could advance our understanding of managerial work in (public) healthcare and how it is influenced by institutional and regulatory factors (including organizational structure or boards supporting the CEO), as well as by organizational features. Finally, we lack data regarding the performances associated with CEOs' use of time.

## Conclusions and practice implications

Our study findings show that executives in pluralistic public healthcare organizations contend with three major problems. The first is how to employ their time wisely and strike a balance between working hours in operational activities and in strategic activities. It would be oversimplistic to think that top managers should focus only their organization’s strategic agenda, as they are oftentimes called to put out fires and respond to stakeholders, show understanding, and involve them in decision making. Interaction in dealing with apparently mundane and operational problems, informal chats, and network building are necessary activities, and a CEO’s activity is necessarily highly fragmented and that it often entails wrestling with day-to-day problems rather than long-term planning issues.

Such complexity and ambiguity should not become an excuse for devoting energy to operational time, however. Making quick decisions is more attractive than designing the future, it is usually what CEOs have studied for and what generates short-term self-realization. Many problems cannot wait indefinitely, whereas change and innovation can – or so it is believed. Though loath to admit so, CEOs may prefer operational time because it is problem-solving time. An incompetent CEO might be able to hide ineffective managerial behavior behind an apparent commitment to operational activities. And such a CEO may be able to devise defensive maneuvers that preclude attendance to managerial work. In such circumstances, the agenda will be based *only* on responding to emergencies under the pressure of stakeholders. The lack of strategy could impede change and ultimately result in paralysis [2, 29].

Related to this issue is delegation. While we do not believe that external support for CEOs in public healthcare organizations is requisite to help them respond to pressures from multiple stakeholders, we feel that building a strong strategic board with complementary capabilities is fundamental. In the context of the Italian NHS, administrative and medical directors may supervise specific aspects of management, for instance, distributing it between strategic board members who deal primary with internal matters (e.g., medical director) and those more engaged with external stakeholders (possibly the CEOs). More broadly, CEOs should delegate responsibility not only to other top managers but also to middle level managers and clinical leaders. This is problematic, however, as it involves developing trust, relinquishing power, and training managerial competences in the physicians lacking them [47]. And some CEOs might not have the willingness or the capacity to do so.

Health organizations urgently need CEOs who can navigate current challenges and future paradigm shifts. There are dramatic changes ahead in the delivery of healthcare services. Research may help to better shape our knowledge about the strategies that CEOs will need to adopt for guiding complex healthcare organizations. This may open new avenues for future research and lay the basis for a much-needed redesign of the training content for CEOs in healthcare organizations.

## Supplementary Information

**Additional file 1: Appendix A.** Details of the survey tool.

## Data Availability

The dataset used for the study is available from the corresponding author on reasonable request.

## Abbreviations

CEO: Chief Executive Officer
NHS: National Health Service

## References

[1] Glouberman S, Mintzberg H (2001). Managing the care of health and the cure of disease–part I: differentiation. Health Care Manag Rev.

[2] Denis JL, Langley A, Rouleau L (2007). Strategizing in pluralistic contexts: rethinking theoretical frames. Hum Relat.

[3] Hoff TJ (1999). The paradox of legitimacy: physician executives and the practice of medicine. Health Care Manag Rev.

[4] Lega F (2012). Beyond rhetoric, inquiry on the essence of strategic management in public healthcare organisations. Int J Clin Leadersh.

[5] Liang Z, Howard P, Wang J, Xu M, Zhao M (2020). Developing senior hospital managers: does ‘one size fit all’? – evidence from the evolving Chinese health system. BMC Health Serv Res.

[6] Mascia D, Piconi I (2013). Career histories and managerial performance of health care CEOs: an empirical study in the Italian National Health Service. Health Care Manag Rev.

[7] Goodall AH (2011). Physician leaders and hospital performance: is there an association?. Soc Sci Med.

[8] Mintzberg H (1973). The Nature of Managerial Work.

[9] Fayol H (1916). General and Industrial Management.

[10] Gulick L, Gulick L, Urwick L (1937). Notes on the theory of organization. Papers on the Science of Administration.

[11] Mintzberg H (1971). Managerial work: analysis from observation. Manag Sci.

[12] Mintzberg H (2009). Managing.

[13] Tengblad S, Vie OE, Tengblad S (2012). Management in practice: overview of classic studies on managerial work. The work of managers: towards a practice theory of management.

[14] Kurke L, Aldrich H (1984). Mintzberg was right: a replication and extension of the nature of managerial work. Manag Sci.

[15] Tengblad S (2006). Is there a ‘New Managerial Work’? A comparison with Henry Mintzberg’s classic study 30 years later. J Manag Stud.

[16] Bandiera O, Hansen S, Prat A, Sadun R (2017). CEO behavior and form performance. Working Paper 23248, National Bureau of Economic Research.

[17] Porter M, Nohria N (2018). How CEOs manage time. Harvard Bus Rev.

[18] Kotter JP (1987). The leadership factor.

[19] Dargie C (2000). Observing chief executives: analysing behaviour to explore cross-sectoral differences. Public Manag Money..

[20] Noordegraaf M, Stewart R (2000). Managerial behaviour research in private and public sectors: distinctiveness, disputes and directions. J Manag Stud.

[21] Korica M, Nicolini D, Johnson B (2017). In search of ‘Managerial Work’: past, present and future of an analytical category. Int J Manag Rev.

[22] Johnson B, Dobni D (2016). Is managerial work in the public and private sectors really “Different”? A comparative study of managerial work activities. Int J Public Adm.

[23] Jarzabkowski P, Fenton E (2006). Strategizing and organizing in pluralistic contexts. Long Range Plan.

[24] Denis JL, Ferlie E, Van Gestel N (2015). Understanding Hybridity in Public Organizations. Public Adm.

[25] Sarto F, Veronesi G (2016). Clinical leadership and hospital performance: assessing the evidence base. BMC Health Serv Res.

[26] Denis JL, Lamothe L, Langley A (2001). The Dynamics of Collective Leadership and Strategic Change in Pluralistic Organizations. Acad Manag J.

[27] Denis JL, Langley A, Cazale L (1996). Leadership and strategic change under ambiguity. Organ Stud.

[28] Jarzabkowski P, Balogun J (2009). The practice and process of delivering integration through strategic planning. J ManagStud.

[29] Cuccurullo C, Lega F (2013). Effective strategizing practices in pluralistic settings: the case of academic medical centers. J Manag Gov.

[30] Gilmartin MJ, D'Aunno TA (2007). Leadership Research in Healthcare. Acad Manag Ann.

[31] Clarke JM, Waring J, Bishop S, Hartley J, Exworthy M, Fulop NJ, Ramsay A, Roe B (2021). The contribution of political skill to the implementation of health services change: a systematic review and narrative synthesis. BMC Health Serv Res.

[32] Rainey HG, Bozeman B (2000). Comparing public and private organizations: empirical research and the power of the ‘a priori’. J Public Adm Res Theory.

[33] Orazi DC, Turrini A, Valotti G (2013). Public sector leadership: new perspectives for research and practice. Int Rev Adm Sci.

[34] Lega F, De Pietro C (2005). Converging patterns in Hospital Organisation: beyond the professional Bureaucracy. Health Policy.

[35] Lega F, Longo F, Rotolo A (2013). Decoupling the use and meaning of strategic plans in public healthcare. BMC Health Serv Res.

[36] Cinelli G, Gugiatti A, Meda F, Petracca F, CERGAS (2020). La struttura e le attività del SSN. Rapporto OASI 2020.

[37] Sartirana M, Prenestini A, Lega F (2014). Medical management: hostage to its own history? The case of Italian clinical directors. Int J Public Sector Manag.

[38] Fattore G, Longo F, Sartirana M, CERGAS (2013). Il curriculum vitae dei Direttori Generali. Rapporto OASI 2013.

[39] Jick TD (1979). Mixing qualitative and quantitative methods: triangulation in action. Adm Sci Q.

[40] Kvale S, Brinkmann S. Learning the Craft of Qualitative Research Interviewing. California: SAGE Publications; 2009.

[41] Weiss RS (1994). Learning from strangers: the art and method of qualitative interview studies.

[42] Locke K, Golden-Biddle K, Feldman MS (2008). Perspective-making doubt generative: rethinking the role of doubt in the research process. Organ Sci.

[43] Mantere S, Kekokivi M (2013). Reasoning in organization science. Acad Manag Rev.

[44] Alvesson M, Kärreman D (2007). Constructing mystery: empirical matters in theory development. Acad Manag Rev.

[45] Braun V, Clarke V (2006). Using thematic analysis in psychology. Qual Res Psychol.

[46] Saldana J (2012). The coding manual for qualitative researchers.

[47] Sartirana M, Currie G, Noordegraaf M (2019). Interactive identity work of professionals in management: a hospital case study. Public Manag Rev.

