# Supplementary material for: Dealing with pluralism: the managerial work of CEOs in Italian public healthcare organizations
Source: BMC Health Serv Res. 2022 Oct 1;22:1222. doi: 10.1186/s12913-022-08567-1 (PMC9526245; doi:10.1186/s12913-022-08567-1)
Supplement: Supplementary file 1 — Additional file 1: Appendix A. Details of the survey tool. [file 12913_2022_8567_MOESM1_ESM.docx]

**Appendix A - Details of the survey tool**

An online survey was sent via email to the personal assistants of CEOs in Italian public healthcare organizations. The questionnaires were sent in two waves, one in April 2019 and the other in May 2019.

*Online questionnaire 1 [filled in by the CEO’s personal assistant]*

- How many hours did the CEO devote to each of the activities listed below in the week from 4 March to 10 March 2019? The same item was repeated for the next three weeks:
  - In-person meetings
  - Remote meetings
  - Individual work (tracked in diary entries)
  - Public events
  - Business trips
  - Continuing professional education
  - Family and private life (tracked in diary entries)
- Please estimate the total number of hours in meetings from 4 to 10 March 2019 as tracked in the previous question and calculate the percentage of time spent with each type of stakeholder. The same item was repeated for the next three weeks:
  - Regional government administrators
  - Strategic board members
  - CEOs from other healthcare organizations
  - Local communities
  - Union representatives
  - Patient associations
  - Organizational ancillary services
  - Healthcare professionals
  - Pharma and medical technology companies
  - Press / media
  - Other stakeholders: please specify

*Online questionnaire 2 [filled in by CEOs]*

- Please estimate the total number of hours in meetings with each type of stakeholder in March 2019, as shown in the chart attached (data provided in the first questionnaire) and calculate the percentage of time devoted to the strategic agenda (“time directly connected with the goals of the mandate and the organization’s medium or long range objectives, as defined in strategic documents”), and that devoted to handling unfolding issues and dealing with have-to-dos (“operational time or time related to unplanned requests by stakeholders”):
  - Regional government administrators
  - Strategic board members
  - CEOs from other healthcare organizations
  - Local communities
  - Union representatives
  - Patient associations
  - Organizational ancillary services
  - Healthcare professionals
  - Pharma and medical technology companies
  - Press / media
  - Other
